# Metabolic Influence of *S. boulardii* and *S. cerevisiae* in Cross-Kingdom Models of *S. mutans* and *C. albicans*

**DOI:** 10.3390/jof11040325

**Published:** 2025-04-19

**Authors:** Ting Li, Xingyi Lu, Yan Wu, Tongtong Wu, Jin Xiao

**Affiliations:** 1 Eastman Institute for Oral Health, University of Rochester Medical Center, Rochester, NY 14642, USA; ting_li@urmc.rochester.edu (T.L.); xingyi_lu@urmc.rochester.edu (X.L.); yan_wu@urmc.rochester.edu (Y.W.); 2 College of Laboratory Medicine, Chongqing Medical University, Chongqing 400016, China; 3 Department of Biostatics and Computational Biology, University of Rochester Medical Center, Rochester, NY 14620, USA; tongtong_wu@urmc.rochester.edu

**Keywords:** *Saccharomyces boulardii*, *Saccharomyces cerevisiae*, *Streptococcus mutans*, *Candida albicans*, metabolic influence, microbial interactions

## Abstract

Recent studies highlight the potential of *Saccharomyces* species as probiotics due to their ability to modulate microbial interactions and reduce cariogenic activity, yet the underlying metabolic mechanisms remain unclear. This study investigates the cross-kingdom metabolic effects of *Saccharomyces boulardii* and *Saccharomyces cerevisiae* on the metabolic processes of *Streptococcus mutans* and *Candida albicans* using a metabolomics-based approach. Untargeted LC-MS/MS analysis was conducted to assess metabolites in a planktonic model, followed by metabolomic profiling and pathway analysis to identify key metabolic alterations. The results revealed that *S. boulardii* and *S. cerevisiae* demonstrated metabolic regulatory effects on *S. mutans* and *C. albicans*. Specifically, *S. boulardii* down-regulated 262 metabolites and up-regulated 168, while *S. cerevisiae* down-regulated 265 metabolites and up-regulated 168. Both yeast species down-regulated carbohydrate and amino acid metabolism in *S. mutans* and *C. albicans*, resulting in reduced biomolecule synthesis and a less acidic environment. *S. boulardii* and *S. cerevisiae* also up-regulated certain metabolic processes, including purine metabolism, suggesting a compensatory mechanism for nucleotide synthesis. Notably, dual regulatory effects were observed, where specific metabolites were simultaneously up-regulated and down-regulated, indicating complex metabolic crosstalk. These findings suggest that both *S. boulardii* and *S. cerevisiae* modulate microbial metabolism through a shared mechanism, offering potentials for dental caries prevention.

## 1. Introduction

Dental caries is a multifactorial disease caused by acid-producing microorganisms, including bacteria and fungi, that demineralize tooth enamel, leading to cavities [1]. The process begins with the formation of bacterial biofilms, where fermentable carbohydrates are metabolized into acids. This process lowers the pH in the oral environment, gradually dissolving tooth minerals and contributing to enamel demineralization [2]. *Streptococcus mutans* and *Candida albicans* interact synergistically to enhance biofilm formation and increase acid production, accelerating the pathogenesis of dental caries [3]. *S. mutans* produces extracellular polysaccharides that aid *C. albicans* adhesion, while *C. albicans* boosts *S. mutans* virulence by stimulating its metabolic activity, creating a highly cariogenic environment [4].

Treatment for dental caries primarily involves invasive restoration [5]. Probiotics have demonstrated potential as an adjunctive therapy for caries prevention by competing with cariogenic bacteria, inhibiting their adhesion and proliferation in the oral cavity, producing antimicrobial compounds, and modulating the host’s immune response [6]. Probiotic yeasts uniquely inhibit biofilm formation, neutralize acids, and restore microbial balance, suppressing *S. mutans* and reducing cariogenic activity, making them a promising alternative or adjunctive therapy for caries prevention and management [7].

*Saccharomyces* species have been explored as a probiotic for their antimicrobial, anti-inflammatory, and immunomodulatory properties in various diseases, including dental caries and gastrointestinal disorders [8,9,10]. *Saccharomyces* can inhibit cariogenic bacteria such as *S. mutans* by competing for adhesion sites, producing antimicrobial compounds, and modulating the oral microbiome [11,12]. Additionally, *Saccharomyces* is capable of reinforcing gut barrier integrity and regulating immune responses, making it effective in managing inflammatory diseases, antibiotic-associated diarrhea, and *Clostridium difficile* infections [13,14]. In addition, *Saccharomyces* exerts its probiotic effects through mechanisms such as pathogen exclusion, secretion of bioactive metabolites, and modulation of host immune responses, contributing to overall microbial balance and health. These properties make *Saccharomyces* a promising adjunct in both oral and systemic disease management.

Among probiotic yeasts, *Saccharomyces boulardii* and *Saccharomyces cerevisiae* have demonstrated significant metabolic regulatory effects that contribute to both oral and overall health. *S. boulardii*, a well-studied probiotic yeast, is widely used to prevent and treat gastrointestinal disorders [15,16]. *S. boulardii* functions by inhibiting pathogen adhesion, neutralizing bacterial toxins, and modulating immune responses to reduce intestinal inflammation. *S. cerevisiae*, the most prominent yeast used as a feed additive, has also shown probiotic potential by promoting gut microbiota balance and improving digestion [17]. *S. cerevisiae* contributes to gut health by producing antimicrobial peptides, enhancing short-chain fatty acid production, and modulating the host immune system, making it beneficial for conditions like irritable bowel syndrome and colitis [18]. Both yeasts contribute to maintaining microbial homeostasis, making them promising candidates for caries prevention and oral health promotion.

Our previous research confirmed the effects of *S. boulardii* and *S. cerevisiae* on the cross-kingdom interactions between cariogenic *S. mutans* and *C. albicans* [11]. However, the precise mechanisms underlying their metabolic regulatory effects remain unclear. In this study, we employed untargeted metabolomics analysis using LC-MS/MS to investigate metabolic changes within the multi-species planktonic model. This approach allowed us to explore how *S. boulardii* and *S. cerevisiae* regulate the metabolism of *S. mutans* and *C. albicans*, providing insights into the metabolomic mechanisms of using probiotic yeast to prevent dental caries.

## 2. Materials and Methods

### 2.1. Bacteria and Yeast Strains and Starter Preparation

The strains *S. mutans* UA159, *C. albicans* SC5314, and *Saccharomyces (S. boulardii* ATCC MYA796 and *S. cerevisiae* ATCC 204508) were purchased from ATCC (Manassas, VA, USA) and recovered from frozen stock and cultivated on specific media: blood agar (TSA with sheep blood, Thermo Scientific^TM^, Waltham, MA, USA, catalog number R01202), YPD agar (BD Difco^TM^, San Jose, CA, USA, catalog number 242720), and yeast mold agar (BD Difco^TM^, Franklin Lakes, NJ, USA, catalog number 271210), respectively. After 48 h incubation, *S. mutans* was transferred to TSBYE broth (3% Tryptic Soy, 0.5% Yeast Extract Broth, BD Bacto^TM^ 286220 and Gibco^TM^ 212750) with 1% glucose; *C. albicans*, *S. boulardii*, and *S. cerevisiae* were cultured in YPD broth (BD Difco^TM^, 242820). The next morning, each overnight culture was diluted with fresh broth, followed by a 3–4 h incubation to reach the mid-exponential phase with desirable optical density (OD), and adjusted to starting concentrations for further experiments.

### 2.2. Planktonic Model

**Figure 1:**
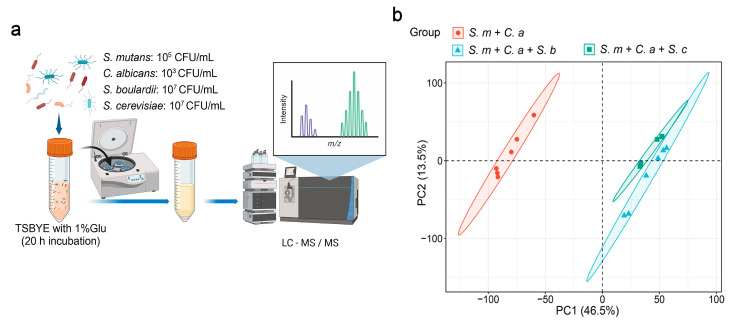
The untargeted metabolomics analysis of the effects of *Saccharomyces* on *S. mutans*–*C. albicans*. (**a**) A schematic representation of the process of a planktonic model (created with BioRender.com). Dual-species and multi-species conditions of *Streptoccocus mutans* (10^5^ CFU/mL), *Candida albicans* (10^3^ CFU/mL), and *Saccharomyces* (*S. boulardii* or *S. cerevisiae,* 10^7^ CFU/mL) in 10 mL of TSBYE broth supplemented with 1% glucose for 20 h. (**b**) Principal component analysis (PCA) two-dimensional scores plot from the untargeted metabolomics analysis, with each dot representing a biological sample.

Initial concentrations were set at 10^5^ CFU/mL for *S. mutans* and 10^3^ CFU/mL for *C. albicans* to replicate high-risk caries conditions, while *Saccharomyces* species were set at 10^7^ CFU/mL based on their inhibitory effects. Dual-species cultures (*S. mutans* and *C. albicans*) and multi-species cultures (*S. mutans*, *C. albicans*, and either *S. boulardii* or *S. cerevisiae*) were cultivated in 10 mL of TSBYE broth supplemented with 1% glucose (5% CO_2_, 37 °C) for 20 h. After incubation, supernatants were collected by centrifugation and stored at −80 °C for LC-MS/MS untargeted metabolomics analysis to evaluate the effects of *Saccharomyces* on the metabolism (Figure 1a).

### 2.3. Liquid Chromatography-Tandem Mass Spectrometry (LC-MS/MS)

Metabolomics analysis was performed using a Thermo Vanquish HPLC/Orbitrap ID-X MS (Thermo Fisher Scientific, Waltham, MA, USA) [19]. Samples were thawed, aliquoted, and extracted with ice-cold methanol, followed by vortexing and centrifugation. Supernatants were dried under nitrogen and reconstituted in HPLC mobile phases, with quality control (QC) samples prepared similarly. Analysis involved four LC/MS experiments, utilizing reversed-phase C18 and HILIC chromatography in both positive and negative ionization modes on an Orbitrap ID-X mass spectrometer, scanning from m/z 70–1000 at a resolution of 120,000.

### 2.4. Statistical Analysis

Data from the raw outputs were processed using Thermo Scientific’s Compound Discoverer (version 3.4). Metabolites with *p*-value < 0.05 after the False Discovery Rate (FDR) correction were characterized as statistically significant.

### 2.5. Pathway Analysis

Metabolite Pathway analysis (MetPA) was performed by MetaboAnalyst 6.0, shedding light on the biological mechanisms and biochemical pathways which were involved and altered by *Saccharomyces*. The names of the statistically significant metabolites were imported as input, the hypergeometric test was chosen as the enrichment method, the relative betweenness centrality was preferred for topological analysis, while the specific libraries were the selected as references. *Streptococcus pyogenes* M1 476 (serotype M1) (KEGG) and *C. albicans* (KEGG) were selected as the pathway library of *S. mutans* and *C. albicans*, respectively. Cytoscape software 3.10.3 was then used to draw the pathway network.

## 3. Results

### 3.1. Impact of S. boulardii on S. mutans Metabolomics

The quality of the metabolic profiling data was evaluated through a principal component analysis (PCA) of all replicated samples. The clustering of samples in PCA plots indicated distinct global metabolomic profiles between groups (Figure 1b).

**Figure 2:**
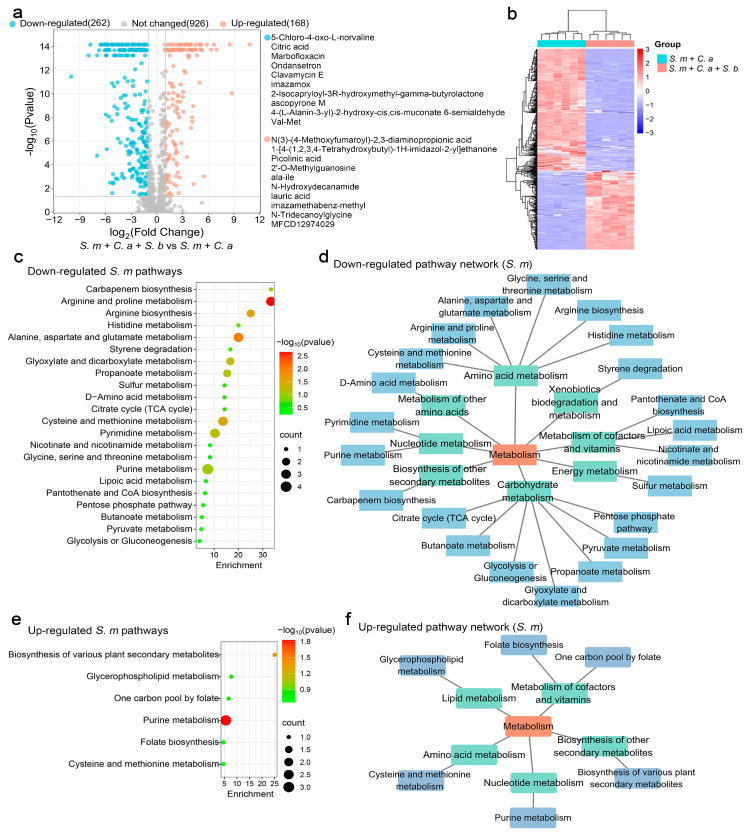
The functional analysis of *S. mutans* metabolites regulated by *S. boulardii.* (**a**) A volcano plot showing 168 up-regulated metabolites (adjusted *p* < 0.05, log2 FC > 1) and 262 down-regulated metabolites (adjusted *p* < 0.05, log2 FC < −1) in the multi-species model with *S. boulardii* added. (**b**) A clustering heatmap illustrating the classification of metabolites regulated by *S. boulardii* in planktonic models. The rows (metabolites) and columns (samples) are clustered separately, with raw data normalized to Z-scores. The mapping grids are color-coded according to their Z-scores. (**c**) The analysis of down-regulated metabolic pathways using the web-based MetaboAnalyst 6.0, based on *S. mutans* pathway libraries. (**d**) Down-regulated metabolic pathway networks in *S. mutans*. (**e**) The analysis of up-regulated metabolic pathways based on *S. mutans* pathway libraries. (**f**) Up-regulated metabolic pathway networks in *S. mutans*.

The analysis revealed that the addition of *S. boulardii* significantly modulated the production of 430 metabolites in *S. mutans*, with 262 metabolites down-regulated and 168 up-regulated. The top ten most significantly up-regulated and down-regulated metabolites are listed (Figure 2a). A clustering heatmap illustrated clear distinctions in metabolite profiles between dual-species and multi-species models, underscoring the influence of *S. boulardii* (Figure 2b).

Pathway analysis of the significantly regulated metabolites (adjusted *p*-value < 0.05, log2 (Fold Change) < −1) showed substantial down-regulation in 22 metabolic pathways, with arginine and proline metabolism being the most affected (Figure 2c). These pathways are crucial for energy production and biomolecule synthesis, highlighting the impact of *S. boulardii* on metabolic pathways involved in carbohydrate metabolism, amino acid metabolism, nucleotide metabolism, energy metabolism, the metabolism of cofactors and vitamins, and the biosynthesis of other secondary metabolites (Figure 2d). Conversely, six pathways were up-regulated, with purine metabolism being the most significantly enhanced (Figure 2e). These up-regulated pathways were enriched in the metabolism of cofactors and vitamins, biosynthesis of other secondary metabolites, amino acid metabolism, lipid metabolism, and nucleotide metabolism (Figure 2f).

### 3.2. Influence of S. boulardii on C. albicans Metabolomics

**Figure 3:**
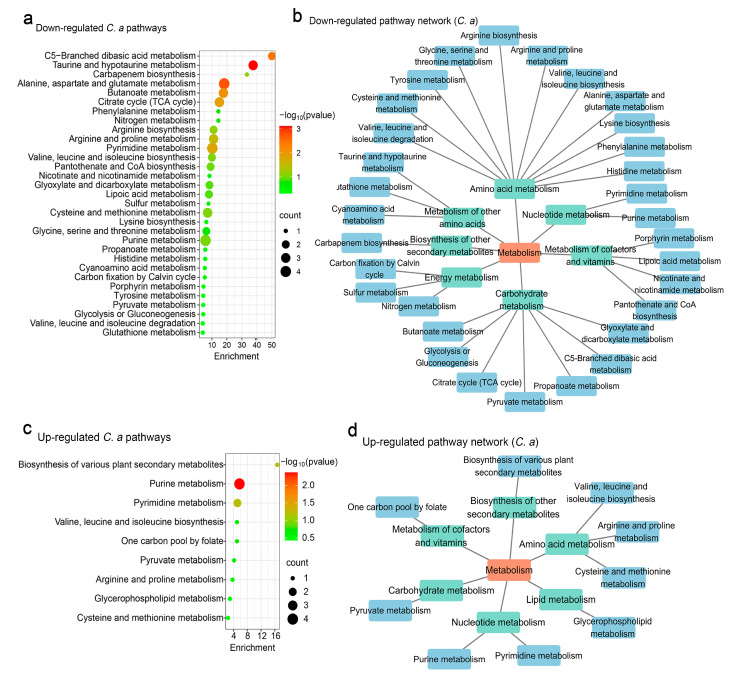
Functional analysis of *C. albicans* metabolites regulated by *S. boulardii.* (**a**) Analysis of down-regulated metabolic pathways using web-based MetaboAnalyst 6.0, based on *C. albicans* pathway libraries. (**b**) Down-regulated metabolic pathway networks in *C. albicans*. (**c**) Analysis of up-regulated metabolic pathways based on *C. albicans* pathway libraries. (**d**) Up-regulated metabolic pathway networks in *C. albicans*.

Pathway analysis of *C. albicans* indicated that *S. boulardii* significantly down-regulated 31 metabolic pathways. Taurine and hypotaurine metabolism were the most affected, with implications for carbohydrate metabolism, amino acid metabolism, the metabolism of cofactors and vitamins, metabolism of other amino acids, energy metabolism nucleotide metabolism, and biosynthesis of other secondary metabolites (Figure 3a,b). These changes highlight the broad metabolic impact of *S. boulardii* on *C. albicans*.

For up-regulated metabolites, nine pathways were identified, with purine metabolism showing the most significant increase (Figure 3c). These pathways enriched processes related to the metabolism of cofactors and vitamins, the biosynthesis of other secondary metabolites, amino acid metabolism, lipid metabolism, nucleotide metabolism, and carbohydrate metabolism, demonstrating the dynamic metabolic shifts induced by *S. boulardii* (Figure 3d).

### 3.3. Effects of S. cerevisiae on S. mutans Metabolism

**Figure 4:**
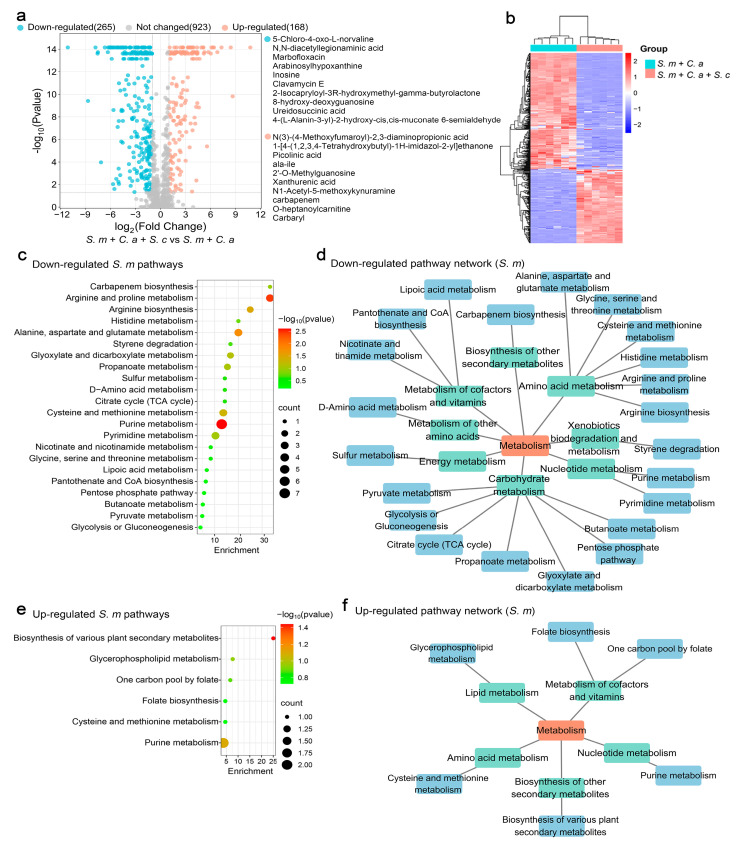
The functional analysis of *S. mutans* metabolites regulated by *S. cerevisiae.* (**a**) A volcano plot showing 168 up-regulated metabolites (adjusted *p* < 0.05, log2 FC > 1) and 265 down-regulated metabolites (adjusted *p* < 0.05, log2 FC < −1) in the multi-species model with *S. cerevisiae* added. (**b**) A clustering heatmap illustrating the classification of metabolites regulated by *S. cerevisiae* in planktonic models. The rows (metabolites) and columns (samples) are clustered separately, with raw data normalized to Z-scores. The mapping grids are color-coded according to their Z-scores. (**c**) The analysis of down-regulated metabolic pathways using the web-based MetaboAnalyst 6.0, based on *S. mutans* pathway libraries. (**d**) Down-regulated metabolic pathway networks in *S. mutans*. (**e**) The analysis of up-regulated metabolic pathways based on *S. mutans* pathway libraries. (**f**) Up-regulated metabolic pathway networks in *S. mutans*.

Similarly, the presence of *S. cerevisiae* led to significant alterations in 433 metabolites in *S. mutans*, with 265 metabolites down-regulated and 168 up-regulated. The most prominent changes are listed, providing insights into the metabolic adjustments (Figure 4a). Heatmap analysis revealed clear differences in altered metabolite profiles, emphasizing the regulatory capacity of *S. cerevisiae* (Figure 4b).

Pathway analysis using *S. mutans* pathway libraries showed that 22 metabolic pathways were significantly affected. Arginine and proline metabolism were the most down-regulated, impacting pathways primarily enriched in carbohydrate, amino acid, and cofactor and vitamin metabolism (Figure 4c,d). For up-regulated metabolites, six pathways were identified, with a notable increase in the biosynthesis of various plant secondary metabolites. This highlights *S. cerevisiae*’s role in modulating crucial metabolic processes, including amino acid, lipid, nucleotide, cofactor, and vitamin metabolism, as well as the biosynthesis of other secondary metabolites (Figure 4e,f).

### 3.4. Modulation of C. albicans Metabolism by S. cerevisiae

**Figure 5:**
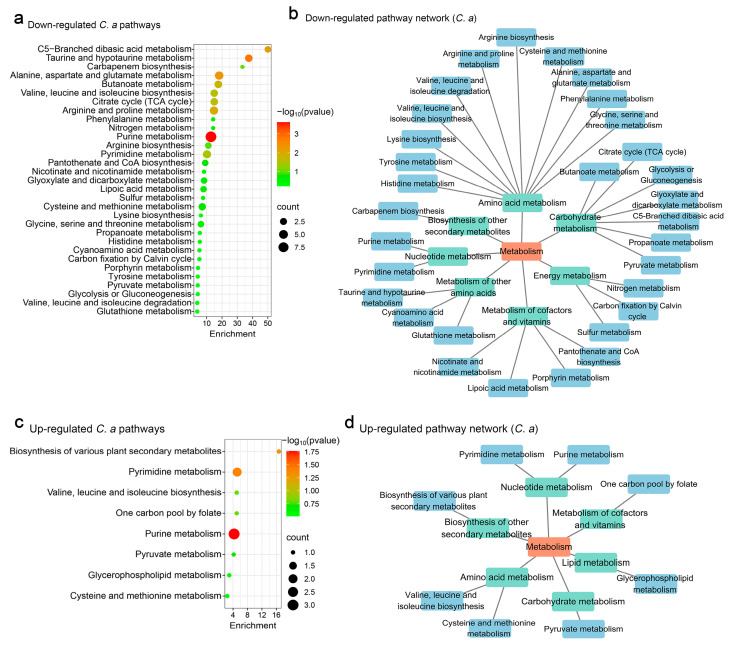
Functional analysis of *C. albicans* metabolites regulated by *S. cerevisiae.* (**a**) Analysis of down-regulated metabolic pathways using web-based MetaboAnalyst 6.0, based on *C. albicans* pathway libraries. (**b**) Down-regulated metabolic pathway networks in *C. albicans*. (**c**) Analysis of up-regulated metabolic pathways based on *C. albicans* pathway libraries. (**d**) Up-regulated metabolic pathway networks in *C. albicans*.

In *C. albicans*, 31 pathways were down-regulated by *S. cerevisiae*, with purine metabolism notably affected (Figure 5a). These down-regulated pathways were primarily enriched in amino acid metabolism, carbohydrate metabolism, and the metabolism of cofactors and vitamins (Figure 5b). Conversely, eight pathways were up-regulated, including significant enhancements in purine metabolism (Figure 5c). These changes enriched processes associated with lipid metabolism, carbohydrate metabolism, amino acid metabolism, cofactor and vitamin metabolism, nucleotide metabolism, as well as the biosynthesis of other secondary metabolites (Figure 5d).

### 3.5. Cross-Species Metabolic Intersection of S. boulardii and S. cerevisiae

**Figure 6:**
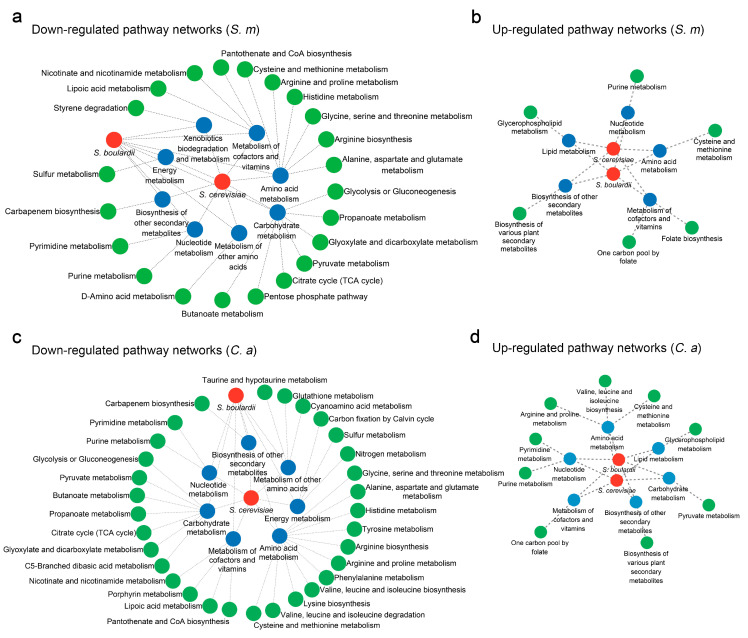
Crosstalk between *S. boulardii* and *S. cerevisiae* on metabolic network of *S. mutans* and *C. albicans.* (**a**) Down-regulated metabolic pathway networks in *S. mutans* caused by *Saccharomyces*. (**b**) Up-regulated pathway networks in *S. mutans* caused by *Saccharomyces*. (**c**) Down-regulated pathway networks in *C. albicans* caused by *Saccharomyces*. (**d**) Up-regulated pathway networks in *C. albicans* caused by *Saccharomyces*.

Using Cytoscape to analyze the metabolic regulatory networks of *S. boulardii* and *S. cerevisiae*, we observed a significant overlap in their regulated classifications and pathways. In *S. mutans*, both yeasts down-regulated metabolism related to nutrient supply and microbial metabolic processes, including carbohydrate metabolism, amino acid metabolism, metabolism of other amino acids, nucleotide metabolism, energy metabolism, the metabolism of cofactors and vitamins, and the biosynthesis of secondary metabolites, collectively affecting 22 metabolic pathways (Figure 6a). Conversely, their up-regulated metabolites were involved in the metabolism of cofactors and vitamins, the biosynthesis of secondary metabolites, amino acid metabolism, lipid metabolism, and nucleotide metabolism, up-regulating six metabolic pathways (Figure 6b).

Similarly, *S. boulardii* and *S. cerevisiae* demonstrated identical metabolic regulation in *C. albicans*. They down-regulated carbohydrate metabolism, amino acid metabolism, the metabolism of cofactors and vitamins, the metabolism of other amino acids, energy metabolism, nucleotide metabolism, and the biosynthesis of other secondary metabolites, affecting 31 metabolic pathways (Figure 6c). The up-regulated metabolites were associated with lipid metabolism, carbohydrate metabolism, amino acid metabolism, cofactor and vitamin metabolism, nucleotide metabolism, and the biosynthesis of other secondary metabolites, encompassing eight metabolic pathways across six categories (Figure 6d).

However, pathway enrichment analysis revealed that some metabolic pathways were simultaneously up-regulated and down-regulated by *Saccharomyces*. To distinguish pathways with both regulatory trends, we further analyzed the localization and function of regulated metabolites throughout the metabolic process (with up-regulation highlighted in red and down-regulation in blue).

Both *S. boulardii* and *S. cerevisiae* exhibited identical regulatory effects on cysteine and methionine metabolism in *S. mutans* and *C. albicans*. Specifically, they up-regulated L-Methionine while down-regulating 2-Oxobutanoate, O-Acetyl-L-serine, and Pyruvate (Figure S1a,b). In the purine metabolism of *S. mutans*, both *Saccharomyces* co-up-regulated xanthine and adenine while down-regulating guanine, xanthosine, deoxyinosine, and AMP. However, *S. boulardii* also up-regulated deoxyguanosine, whereas *S. cerevisiae* down-regulated guanosine, inosine, and hypoxanthine (Figure S2a). The purine metabolism of *C. albicans* largely mirrored that of *S. mutans*, with the additional co-up-regulation of 3’,5’-Cyclic GMP by both yeasts, while *S. cerevisiae* also down-regulated Allantoate and Urate (Figure S2b).

Furthermore, some pathways were enriched repeatedly in both the up-regulated and down-regulated metabolic pathways of *C. albicans*. In pyrimidine metabolism, *S. boulardii* and *S. cerevisiae* simultaneously up-regulated Cytosine while down-regulating Deoxycytidine, Cytidine, Pseudouridine, and N-Carbamoyl-L-aspartate. Additionally, *S. boulardii* uniquely up-regulated Thymine in this pathway (Figure S3a). In pyruvate metabolism, both yeasts up-regulated alpha-Isopropylmalate while down-regulating pyruvate (Figure S3b). In valine, leucine, and isoleucine biosynthesis, *S. boulardii* and *S. cerevisiae* similarly up-regulated alpha-Isopropylmalate while down-regulating 2-Oxobutanoate and Pyruvate. Moreover, *S. cerevisiae* specifically down-regulated (2R,3S)-3-Isopropylmalate (Figure S3c). Additionally, *S. boulardii* contributed to the down-regulation of arginine and proline metabolism in *C. albicans* by down-regulating L-Proline, 4-Guanidinobutanoate, and L-Glutamate (Figure S3d).

## 4. Discussion

### 4.1. Overview and Highlights

The high prevalence of dental caries is strongly linked to the presence of *Streptococcus mutans* and *Candida albicans*, which contribute to biofilm formation, acid production, and enamel demineralization [20]. Recent studies have investigated the anti-caries potential of probiotics by modulating microbial interactions and reducing cariogenic activity [21]. Our previous research demonstrated that *Saccharomyces boulardii* and *Saccharomyces cerevisiae* create a less cariogenic environment by maintaining a neutral pH and suppressing *C. albicans* growth [11]. In this study, we employed a planktonic model to investigate the cross-kingdom regulatory effects of *S. boulardii* and *S. cerevisiae* on the metabolic processes of *S. mutans* and *C. albicans*, using a metabolomics-based approach to gain insights into their mechanistic influence.

This study highlights the significant and consistent influence of *S. boulardii* and *S. cerevisiae* on the metabolic pathways of *S. mutans* and *C. albicans*. These yeasts modulate multiple metabolites across various pathways, emphasizing their potential role in managing microbial dysbiosis. Comprehensive pathway analysis and the identification of dual regulatory trends provide valuable insights into their metabolic interplay with microbial species. These findings establish a foundation for future research into probiotic applications and microbial ecology.

### 4.2. Metabolic Effects of S. boulardii and S. cerevisiae

Half of the top ten metabolites that were up-regulated and down-regulated by *S. boulardii* and *S. cerevisiae* are identical. Among these, up-regulated N(3)-(4-Methoxyfumaroyl)-2,3-diaminopropionic acid may exhibit antimicrobial properties by potentially inhibiting bacterial growth through enzyme inhibition [22]. Picolinic acid, known for its metal-chelating properties, can inhibit microbial growth by depriving bacteria and fungi of essential metal ions [23]. Similarly, the identical down-regulated metabolites—5-chloro-4-oxo-L-norvaline, marbofloxacin, and clavamycin E—are also associated with antibacterial or antifungal activity [24,25,26].

These observations may result from a multifaceted process involving environmental modification by the yeasts, context-dependent microbial responses, immune system modulation, and microbial dynamics, all of which contribute to maintaining homeostasis. It is also plausible that the changes observed arise primarily from yeast-driven alterations in nutrient availability, metabolic by-products, or competitive inhibition. Thus, attributing these shifts solely to regulatory mechanisms may oversimplify the complexity of inter-microbial interactions within a cross-kingdom in vitro model.

To further investigate these metabolic alterations, pathway enrichment analysis was performed on all differential metabolites (adjusted *p* < 0.05, |log2 FC| > 1). Our findings reveal a consistent impact of *S. boulardii* and *S. cerevisiae* on the metabolic activity of *S. mutans* and *C. albicans*. In *S. mutans*, both yeasts significantly modulated a broad spectrum of metabolites, with a notable consistency in the affected pathways. They down-regulated key metabolic pathways related to nutrient processing, including carbohydrate and amino acid metabolism, impacting 22 pathways overall. This suppression led to reduced energy production and biomolecule synthesis, particularly in arginine and proline metabolism. Conversely, both yeasts up-regulated six key metabolic classifications, notably purine metabolism, suggesting a compensatory mechanism to maintain nucleotide synthesis.

Similarly, in *C. albicans*, *S. boulardii* and *S. cerevisiae* down-regulated 31 metabolic pathways, including those critical for energy and amino acid metabolism. They also up-regulated purine metabolism and enhanced six key metabolic classifications, mirroring the effects observed in *S. mutans*. The overlapping regulatory patterns suggest a shared mechanism of action by these yeasts, highlighting their potential for therapeutic applications in microbial community management.

### 4.3. Dual Regulatory Effects on Metabolic Pathways

Interestingly, our findings indicate that *S. boulardii* and *S. cerevisiae* can simultaneously up-regulate and down-regulate specific metabolic pathways, reflecting the complexity of microbial interactions. For example, in cysteine and methionine metabolism, the up-regulation of L-methionine alongside the down-regulation of 2-oxobutanoate and pyruvate suggests finely tuned control over sulfur amino acid pathways [27]. Similarly, in purine metabolism, the concurrent up-regulation of xanthine and adenine, along with the down-regulation of guanine and AMP, points to a balanced modulation of nucleotide synthesis and degradation [28].

The up-regulation of metabolites such as xanthine and adenine in *S. mutans* and *C. albicans* may be linked to enhanced nucleic acid synthesis and repair, supporting cellular survival and maintenance [29]. Conversely, the down-regulation of pyruvate and 2-oxobutanoate suggests a reduction in energy production and amino acid biosynthesis, potentially limiting the growth of these pathogens [30]. Previous host–microbe interaction studies suggest that the metabolic shifts induced by *S. boulardii* may contribute to immune modulation, further influencing microbial survival [31]. In vivo experiments also highlight the capacity of probiotics to promote beneficial microbiota balance while suppressing pathogen proliferation through metabolic regulation [32]. This dual regulation underscores the ability of these yeasts to fine-tune metabolic pathways, optimizing microbial growth and survival in diverse environments. Such insights are crucial for understanding microbial resilience and adaptability.

### 4.4. Limitations

Despite these promising findings, several limitations must be considered. Firstly, the precise molecular mechanisms by which *S. boulardii* and *S. cerevisiae* influence metabolic pathways of *S. mutans* and *C. albicans* remain unclear. Further studies incorporating proteomics or transcriptomics are needed to provide deeper insights. Additionally, while our in vitro results indicate significant metabolic effects of *S. boulardii* and *S. cerevisiae*, the in vivo relevance in human or animal models requires further investigation. Environmental factors, such as interactions with host microbiota, were not fully explored and may significantly influence outcomes. Moreover, biofilms create a protective environment for pathogenic microorganisms, affecting their metabolic activity. Future research should investigate how *S. boulardii* and *S. cerevisiae* influence biofilm architecture, composition, and persistence to better understand their potential therapeutic role in managing cariogenic biofilms. The metabolic changes observed in co-cultures may be driven by general metabolic activity rather than specific regulatory mechanisms. We aim to address this by incorporating additional experimental groups and conducting longitudinal analyses. Furthermore, future research using targeted knockout strains or isotope tracing will be necessary to distinguish true regulatory responses from broader, non-specific metabolic shifts. Finally, while this study provides valuable data on metabolic regulation, it does not assess the long-term effects or potential side effects of prolonged yeast use, which should be considered in future clinical applications.

## 5. Conclusions

In summary, our findings demonstrate that *S. boulardii* and *S. cerevisiae* significantly affect the metabolic environment of *S. mutans* and *C. albicans*. These probiotic yeasts profoundly influence microbial metabolism, which may contribute to their beneficial effects on oral and gastrointestinal health. By simultaneously up-regulating and down-regulating various metabolic pathways, they offer a multi-targeted approach for controlling pathogenic microbial growth. Future research should focus on elucidating the specific molecular mechanisms underlying these metabolic changes and their broader therapeutic implications.

## Data Availability

All data generated or analyzed during this study are included in this article/Supplementary Material. Further inquiries can be directed to the corresponding author.

## Supplementary Materials

The following supporting information can be downloaded at: https://www.mdpi.com/article/10.3390/jof11040325/s1, Figure S1: Analysis of cysteine and methionine metabolism regulated by *Saccharomyces* (with up-regulation highlighted in red and down-regulation in blue); Figure S2: Analysis of purine metabolism regulated by *Saccharomyces*; Figure S3: Analysis of repeated enrichment pathways mediated by *Saccharomyces* in *C. albicans*.
