# Supplementary material for: Metabolic Influence of S. boulardii and S. cerevisiae in Cross-Kingdom Models of S. mutans and C. albicans
Source: J Fungi (Basel). 2025 Apr 19;11(4):325. doi: 10.3390/jof11040325 (PMC12028775; doi:10.3390/jof11040325)
Supplement: Supplementary file 1 [file jof-11-00325-s001.zip › jof-3518244-supplementary.pdf]

Supplementary figures

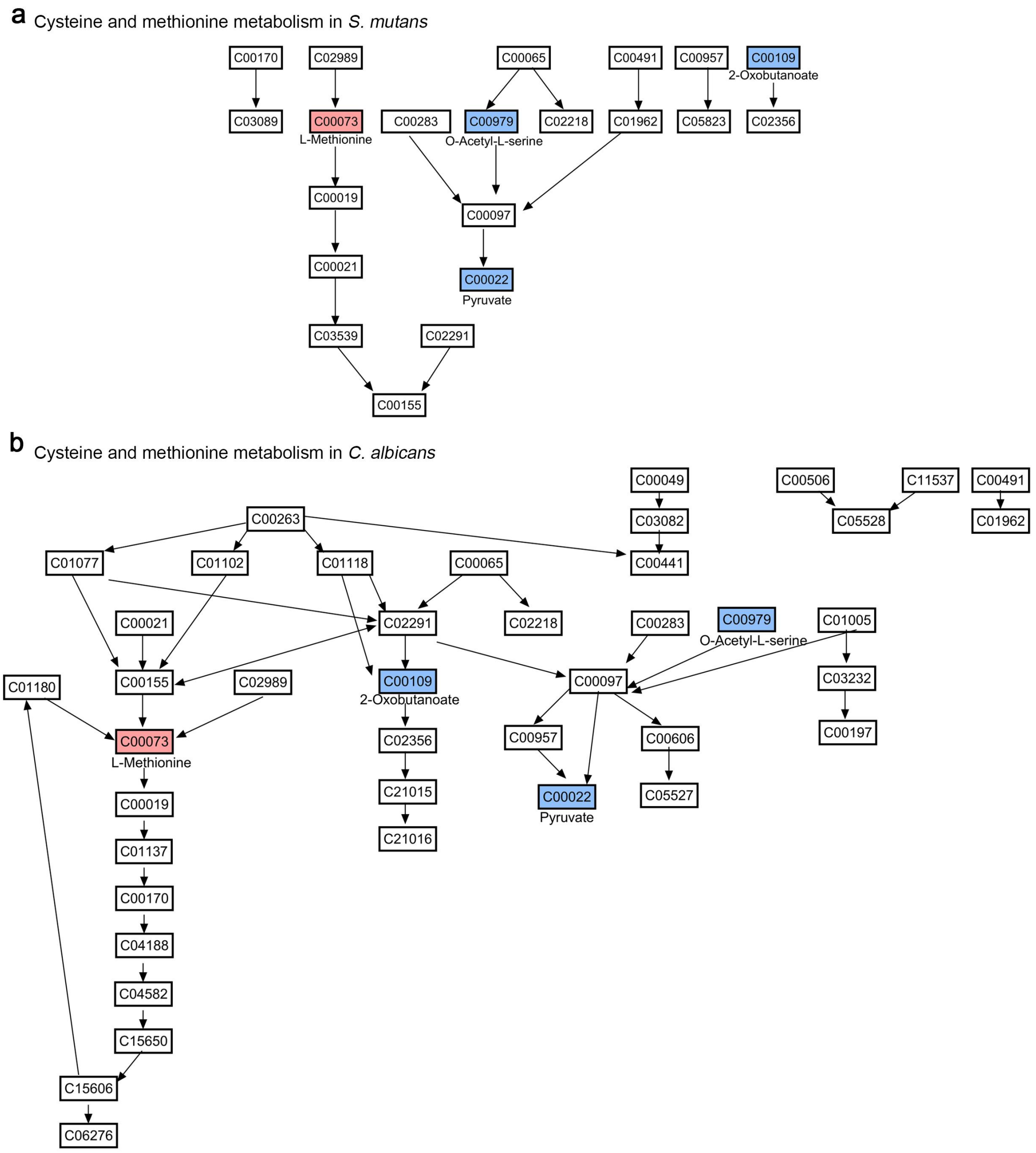

Figure S1. Analysis of cysteine and methionine metabolism regulated by *Saccharomyces* (with up-regulation highlighted in red and down-regulation in blue). (**a**) Identical regulatory effects of *S. boulardii* and *S. cerevisiae* on cysteine and methionine metabolism in *S. mutans*. (**b**) Identical regulatory effects of *S. boulardii* and *S. cerevisiae* on cysteine and methionine metabolism in *C. albicans*.

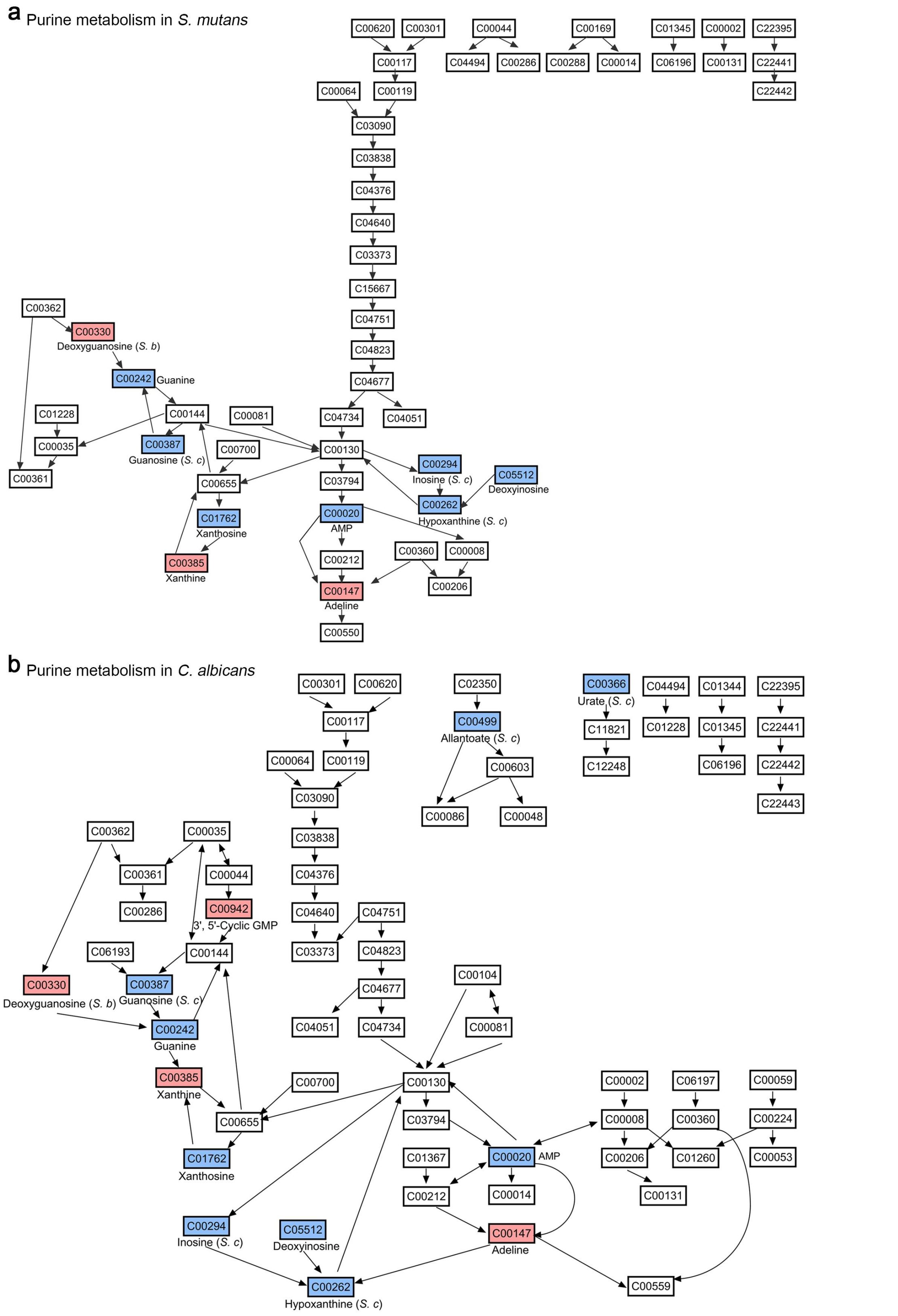

Figure S2. Analysis of purine metabolism regulated by *Saccharomyces*. (**a**) Regulatory effects of *S. boulardii* and *S. cerevisiae* on purine metabolism in *S. mutans*. (**b**) Regulatory effects of *S. boulardii* and *S. cerevisiae* on purine metabolism in *C. albicans*.

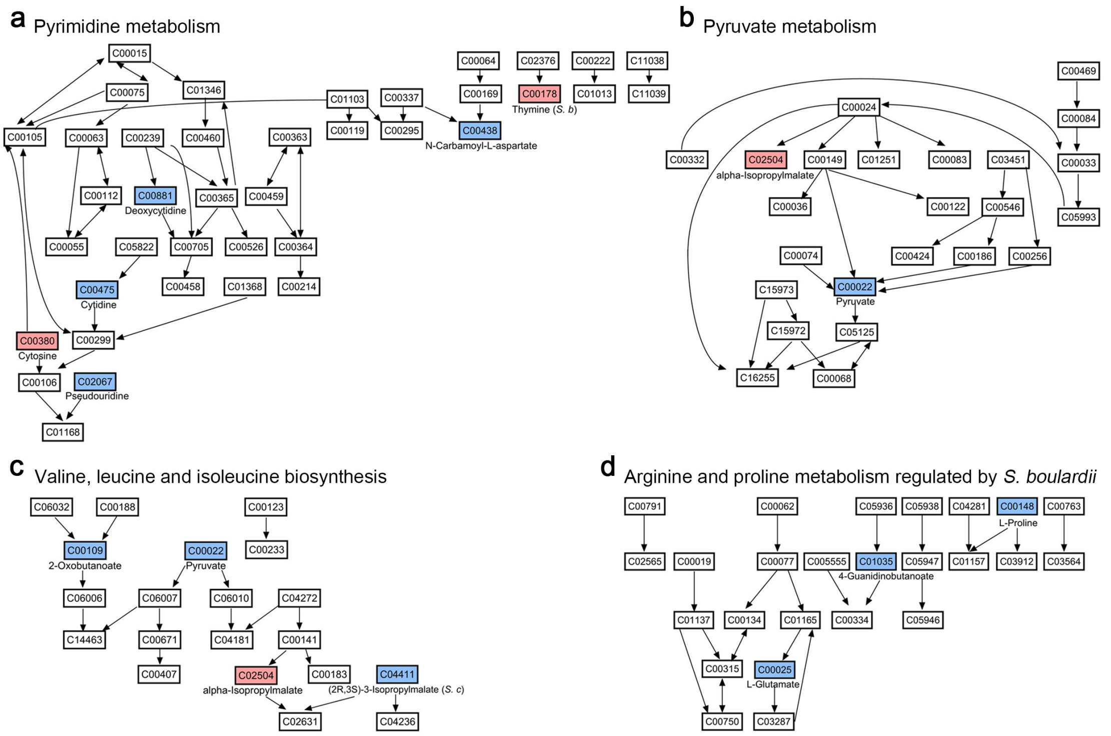

Figure S3. Analysis of repeated enrichment pathways mediated by *Saccharomyces* in *C. albicans*. (**a**) Regulatory effects of *S. boulardii* and *S. cerevisiae* on pyrimidine metabolism. (**b**) Regulatory effects of *S. boulardii* and *S. cerevisiae* on pyruvate metabolism. (**c**) Regulatory effects of *S. boulardii* and *S. cerevisiae* on valine, leucine and isoleucine biosynthesis. (**d**) Regulatory effects of *S. boulardii* on arginine and proline metabolism.
